# Age-Related Trends in Body Composition among Women Aged 20–80 Years: A Cross-Sectional Study

**DOI:** 10.1155/2022/4767793

**Published:** 2022-02-02

**Authors:** Nirmala Rathnayake, Hasanga Rathnayake, Sarath Lekamwasam

**Affiliations:** ^1^Department of Nursing, Faculty of Allied Health Sciences, University of Ruhuna, Galle, Sri Lanka; ^2^Department of Biochemistry, Faculty of Medicine, University of Ruhuna, Galle, Sri Lanka; ^3^Population Health Research Centre, Department of Medicine, Faculty of Medicine, University of Ruhuna, Galle, Sri Lanka

## Abstract

The determinants of body composition are likely to vary geographically due to the diversity of genetic and environmental factors between populations. Age-related trends in body composition in a population help understanding the health issues that are linked with different body compartments. In general, such studies are scarce in the South Asian region and this cross-sectional study examined the age-related trends in body composition in a selected group of healthy women aged 20–80 years in Sri Lanka. The study included randomly selected 784 healthy women aged 20–80 years from the Southern province, Sri Lanka. Women were divided into five age groups: 20–29 years (*n* = 79), 30–39 years (*n* = 144), 40–49 years (*n* = 185), 50–59 years (*n* = 281), and ≥60 years (*n* = 85). Total body bone mineral density (TBBMD, g/cm^2^), total body bone mineral content (TBBMC, g), total body fat mass (TBFM, kg), and total body lean mass (TBLM, kg) were measured with central-type dual-energy X-ray absorptiometry (DXA). Multivariate analysis of variance with Bonferroni post hoc test was performed. The age-related trends of TBBMD and TBBMC were similar with a peak in 40–49-year age group. Between 50 and 59 and ≥60 age categories, TBBMD and TBBMC showed a rapid decline: 16% and 23%, respectively. The rate of TBBMD decline was 0.008 g/cm^2^ per year after 50 years. TBFM increased by 14% between age categories 20–29 and 40–49 years and remained unchanged after 49 years. TBLM increased by 15% between age groups 20–29 and 40–49 years and then decreased by 13% between age categories 50–59 and ≥60 years. Of the 13% decrease in TBLM after 50 years, 9% loss occurred after 59 years. The trends observed help to understand the occurrence of diseases linked with body composition.

## 1. Introduction

Aging is associated with morphological, structural, and functional alterations in the body leading to chronic diseases and physical and mental disability. Furthermore, these bodily changes are associated with loss of independence, frailty, and reduced quality of life (QOL)[1].

The main components of body composition, fat mass (FM) and fat free mass (FFM), are influenced by a multitude of factors such as genetics, environment, ethnicity, age, and sex [2]. The FFM includes lean mass (LM) and bone mineral content (BMC). Many technologies such as dual-energy X-ray absorptiometry (DXA), bioelectrical impedance analysis (BIA), magnetic resonance imaging (MRI), and computed tomography (CT) are used for assessing body composition [3]. Of them, DXA is the most appropriate method recommended for clinical practice since it has a high precision, stable calibration, and low radiation exposure [3]. Quantitative CT and MRI are mainly reserved for research purposes considering the cost and restricted availability.

Different body compartments, despite interacting with each other, behave in an independent manner especially in relation to age. This behavior is linked with both bodily functions and pathological entities at different ages. Generally, FFM peaks between the 4^th^ and 5^th^ decades of life and then declines gradually [4]. FM increases throughout the lifespan, peaks between the 5^th^ and 7^th^ decades of life, and then remains constant or decreases slightly [2]. The loss of LM contributes to the decline in physical function, as well as increasing disability, frailty, and loss of independence [5]. FM is largely dependent on genetic as well as nongenetic factors such as physical inactivity and increased intake of energy-dense foods [6]. Deterioration of both bone material and microarchitecture occurs leading to increased fracture risk with aging. In addition, bone related calcium homeostasis and bone marrow function are affected [7].

The assessment of age-related trends in body composition is beneficial in many ways. The deterioration of FFM with age leads to sarcopenia and osteoporosis, while an increase in FM enhances the risk of cardiovascular disease. These bodily changes are inevitable consequences of aging and can be used to assess functional status, disability, and all-cause mortality [4]. Considering the importance of changes of body composition in both clinical and applied medicine [8, 9], understanding the influence of age on body compartments may help to improve functional capacity and minimize health risks, particularly in older adults.

The studies on body composition dynamics have grown rapidly over the last two decades. Previous studies in this area have mainly involved obese individuals, and specific age groups such as older adults and children [10] and a few on healthy adult women [11, 12]. It is not appropriate to apply data generated elsewhere to local population due to the variations in lifestyle, genetics, and body composition between populations. Further, studies focused on apparently healthy populations in Asian region are scarce in the literature. Previous studies in Sri Lanka have addressed age-related trends in BMD and trabecular bone score [13, 14] and we were unable to find studies that included three main body compartments. Studies on body composition would help establishing normative data for that population and understanding health issues that are linked with different body compartments. Furthermore, they help designing appropriate interventions to reduce the health consequences associated with changes of body composition in the local population. In addition, such studies provide a platform for future studies in this area of research.

Hence, the current study was designed to study the age-related trends in body composition indices measured with DXA and also to develop a reference database that can be used to assess body composition in healthy women in Sri Lanka.

## 2. Materials and Methods

### 2.1. Study Design, Subjects, and Setting

This was a community-based cross-sectional study conducted between 2017 and 2019 in a suburban territory of the Southern province of the country. The research protocol was approved by the Ethical Review Committee of the Faculty of Medicine, University of Ruhuna, Sri Lanka. The participants were made aware of the purpose of the study and written informed consent was obtained prior to data collection.

The latest electoral registers were used to identify community dwelling adult women aged 20 years or above. Participants were recruited using multistage cluster sampling while applying the age-stratified random sampling technique proportionate to the population composition of the region. Subjects for age-based strata were recruited using the systematic random sampling method. During the process, if a woman with exclusion criteria was met, the woman next in the list was considered. Women considered for the study were contacted using the contact numbers obtained from the primary administrative officer in the area.

The sample size for the study was decided utilizing the prevalence (65%) of obesity based on total body fat percentage (TBFP) among 20–60 years aged women residing in a suburban area in Sri Lanka [15], using the formula: *n* = *Z*^2^*P* (1 − *P*)/d^2^ (0.05% error margin and 95% confidence level, *Z* = 1.96; *p* = population proportion, *d* = error margin). After adding 10% for incomplete or missing data, the sample size was 785.

We were able to recruit 784 participants divided into five age groups: 20–29 years (*n* = 79), 30–39 years (*n* = 144), 40–49 years (*n* = 185), 50–59 years (*n* = 281), and ≥60 years (*n* = 95) after excluding those who did not fulfill the selection criteria. The study had power greater than 0.9 in the post hoc power calculation (G^*∗*^ Power 3.1).

The following categories of subjects were excluded from the study after perusing previous medical records, detailed history, and a focused physical examination at the first visit: (1) subjects with diseases which could affect body composition such as hyperthyroidism, hyperparathyroidism, malabsorption, alcohol dependence, chronic inflammatory disease, malignancy, cerebrovascular disease, and hepatic or renal disease; (2) those on medications that could affect body composition such as glucocorticoids, anticonvulsants, bisphosphonates, hormone replacement therapy, thyroxin, and pharmacological doses of calcium and vitamin D; (3) subjects on supervised dietary or exercise programs; (4) those with body deformities (those who presented with skeletal deformities and walking disabilities); (5) women who were pregnant and lactating (from delivery to 2 years minimum); and (6) those with in situ metal prosthesis.

### 2.2. Measurements and Data Collection

Background data including sociodemographic characteristics and gynecologic information were collected using an interviewer-administered questionnaire. The pattern of physical activity (PA) was determined with the short version of the international PA questionnaire (IPAQ) [16]. Daily total energy consumption was obtained from a 24-hour dietary recall (HDR) method. All foods recorded in 24 HDR were converted into grams and then the intake of total energy were analyzed using Indian food composition tables [17] and Sri Lankan food composition tables [18].

Body weight (kg) and height (m) were measured to the nearest 0.1 kg and 0.1 cm, respectively, with a calibrated Stadiometer (NAGATA, Tainan, Taiwan). Circumferences (cm) of waist (WC) and hip (HC) were measured to the nearest 0.1 cm with a plastic measuring tape. Body mass index (BMI, kg/m^2^) and waist to hip ratio (WHR) were calculated. Bone mineral density (g/cm^2^) of total body (TBBMD), total hip (THBMD), total spine (TSBMD), total body BMC (TBBMC, g), total body FM (TBFM, kg), TBFP (%), and total body LM (TBLM, kg) were measured with central-type DXA scanner (Hologic Discovery, Bedford, MA, USA) adhering to the manufacturer's protocols. One technician performed all DXA scans and analyzed them using body composition analysis software provided by the manufacturer. Daily in vitro calibration of the DXA machine, quality control of data, and data analyses were performed by a trained technical officer.

### 2.3. Statistical Analysis

Data were assessed for normality by Kolmogorov Smirnov test. Body composition parameters were expressed as mean (SD) for age groups. Scatter plots of body composition variables were plotted against age and locally weighted scatterplot smoothing lines (Loess) were fitted to observe age-related trends in each variable. Multivariate analysis of variance (MANOVA) with Bonferroni test (post hoc) was used to examine the mean differences between groups. Data were analyzed with SPSS 20.0 version. *p* values <0.05 were considered significant.

## 3. Results

Socioeconomic, clinical and lifestyle characteristics of the participated women are shown in Table 1. Mean (SD) weight and height of the study participants were 56.4 (10.6) kg and 151.2 (5.8) cm, while the mean (SD) WC and HC were 85.3 (10.5) cm and 96.6 (9.7) cm, respectively.

**Figure 1:**
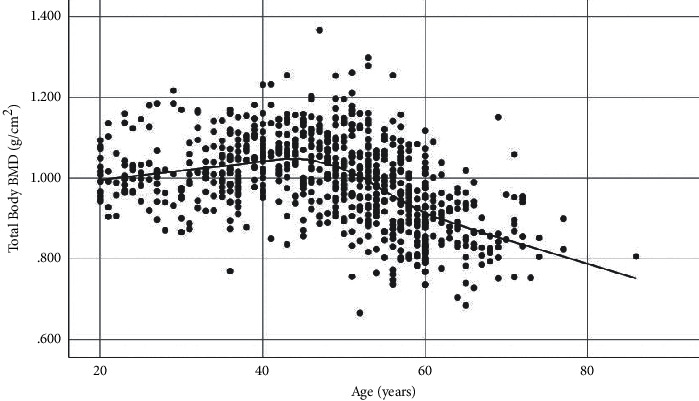
Age-related trend of TBBMD.

**Figure 2:**
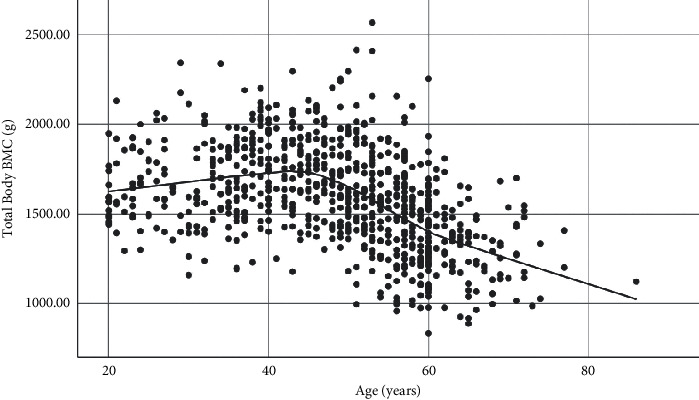
Age-related trend of TBBMC.

TBBMD and TBBMC showed similar trends with increasing age (Figures 1 and 2). The highest mean TBBMD and TBBMC were seen in the 40–49 age group, and both measures gradually decreased afterwards (Figures 1 and 2; Table 2). The increase in TBBMD from 20 to 49 years was significant (4% increase; *p*=0.027), but there was no significant change in TBBMC between the same age range. A rapid decline of both TBBMD and TBBMC was seen between age groups 50–59 and ≥60 years (TBBMD; 16% decrease, *p* < 0.001; TBBMC; 23% decrease, *p* < 0.001). Further, TBBMD declined at the rate of 0.008 g/cm^2^ per year (standard error, 0.012; *p* < 0.001) after 50 years. Similar trend of TBBMD with the aging was observed in BMD at hip and spine (Table 2).

**Figure 3:**
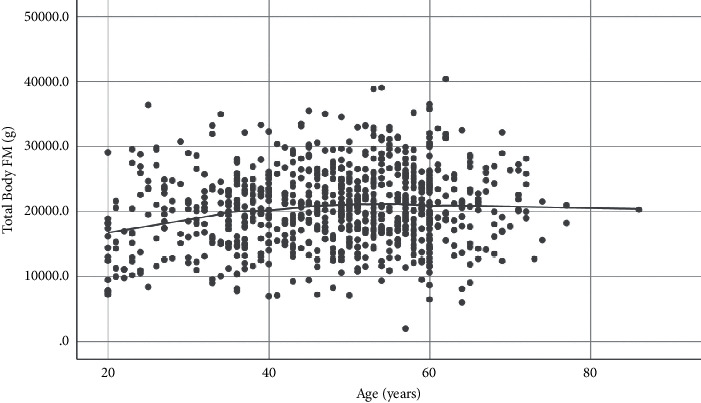
Age-related trend of TBFM.

A significant increase in TBFM (14% increase, *p*=0.012) was seen between 20 and 49 years (Figure 3, Table 2), but the TBFM increase between 50 and 59 and ≥60 years age groups was not significant. Similar trend was seen in the TBFP too (Table 2).

**Figure 4:**
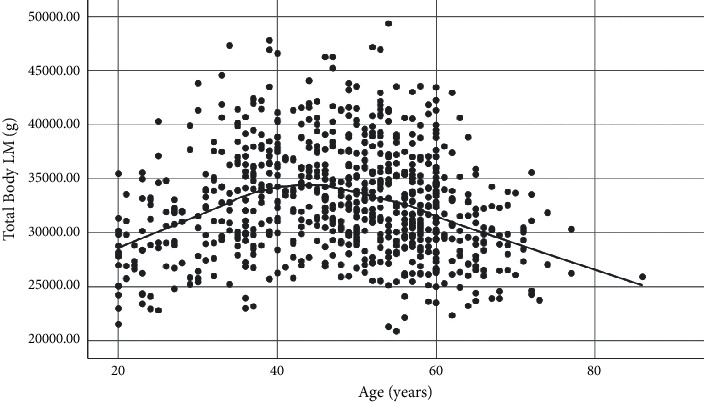
Age-related trend of TBLM.

The highest and lowest mean TBLM were observed in the 40–49 and ≥60 years age groups, respectively (Figure 4, Table 2). TBLM showed a significant increase between 20 and 49 years (15% increase, *p* < 0.001) and then a decrease between 50 and 59 and ≥60 years age groups (13% decrease, *p* < 0.001).

## 4. Discussion

In this study, we report age related trends of body composition measurements of women aged 20 and above in Sri Lanka. The age trends of TBBMD and TBBMC showed a parallel trend with increasing age. Both parameters increased between 20 and 49 years and decreased after 50 years. Both TBFM and TBLM showed significant increase from 20 to 49 years. After 50 years, TBLM decreased while TBFM showed a marginal increase with age.

### 4.1. Changes of BMD and BMC

Local studies on age related trends of body composition based on DXA are scarce. The age at which TBBMD reaches its peak value varies between studies. Although it is believed that BMD reaches its peak between 20 and 30 years, delayed peaking of BMD has been observed in many analyses [19]. According to previous studies, the behaviors of BMD and BMC with age are uniform and predictable and are concordant with our observations. Most of the studies have shown a relatively small increase in BMD and BMC in premenopausal period and a rapid reduction afterwards. A study involving Danish women aged 20–89 years showed both TBBMD and spine BMD to reach the peak values between 30 years and femoral neck and total hip BMDs to reach the peak values between 40 and 49 years [20]. In the same study, there was no significant change in BMDs between the first three decades, but beyond 50 years, an accelerated loss in BMDs in all skeletal sites has been observed. Similarly, a study involving Chinese women showed both TBBMD and spine BMD to reach a peak value between 35 and 39 years and a rapid loss of BMD after 49 years [19]. Further, they observed 19.3% overall TBBMD loss between 35–39 and 70–80 age groups. He et al. reported that the highest bone mass between 41 and 50 years in Chinese Han women and then gradual decrease of both parameters beyond 50 years [21]. Further, ethnic variation in the dynamics of body composition parameters has been observed [22, 23].

Although the exact reasons for the disparity in the behavior of TBBMD, TBBMC, and regional BMDs with age are unclear; environmental, genetic, and epigenetic factors are the plausible explanations. The reduction of bone mass following menopause is considered the main reason for the increased fracture risk seen in women in old age. Low levels of gonadal hormones due to menopause accelerate bone loss in postmenopausal women [24, 25].

### 4.2. Changes of LM

Consistent with our data, cross-sectional and longitudinal studies indicate that the LM reaches its peak value between 4^th^ and 5^th^ decades. Kim et al. observed a greater reduction of LM among Korean women aged >50 years compared to those younger than 50 years [26]. Gába and Přidalová noted the peak LM at 4^th^ decade and slight reduction afterwards in Czech women [27]. The US National Health and Nutrition Examination Survey (NHANES) indicated that LM peaks between 40 and 59 years and declines thereafter [28]. Similar age-related trends in LM have been observed in Western European women [29] and non-Hispanic white women [30]. Furthermore, similar to our findings, a greater loss of LM has been observed during postmenopausal years [31].

The age-related changes in LM may be related to the common pathophysiology linked with mitochondrial activity [32] and hormonal factors such as reduced estrogen [31] and growth hormone and insulin deficiencies [33, 34]. Furthermore, the reduction in energy expenditure and resting metabolic rate with age due to less PA may lead to decrease in LM [35–37]. The age-related trend in LM has been largely consistent irrespective of whether the technology used was DXA [28, 38] or BIA [26]. However, racial and genetic differences can still be expected as some studies found early peak at 3^rd^ decade [39].

### 4.3. Changes of FM

Several longitudinal and cross-sectional studies have reported data similar to the current study. In general, TBFM peaks between the 6^th^ and 7^th^ decades of life [40]. The Fels Longitudinal Study demonstrated that TBFM increases with age in white women [41]. The results of an eight-year longitudinal study showed that TBFM increases in young women (<45 years old) at a lower rate and in women over 45 years at a greater rate [38]. Gába and Přidalová found a significant increase in TBFM as age increased, and the values reached their peak in women over 70 years [27]. Similarly, the NHANES survey found that FM estimated by DXA peaked between the 6^th^ and 7^th^ decades of life, and they declined slightly afterwards [28]. Chumlea et al. [29] showed that TBFM estimated using BIA increased progressively in women between 15 and 98 years. The maximum TBFM was observed between 65 and 74 years, and it declined afterwards. Coin et al. demonstrated the TBFM peak in the 6^th^ decade and TBFM remained constant in the oldest age group [39]. In non-Hispanic white women, TBFM increased from 20 to 59 years, and afterward, it decreased [30]. Though current and many previous studies report the highest TBFM among women >60 years, some studies have reported an early peak of TBFM; Kanaley et al. observed peak TBFM during the perimenopausal years specifically in the 4^th^ to 5^th^ decade and age-related increase in TBFM after menopause [11].

Age trends of TBFM appear to be similar regardless of ethnicity, geographical region, or the method of evaluation such as DXA or BIA. Reduction in energy expenditure due to reduced basal metabolic rate with age [36] and age-related reduced lipoprotein lipase activity [42] may partly explain the increase in TBFM with age. The peak TBFM achieved after menopause is likely to be related to hypoestrogenic effects [43, 44].

### 4.4. Implications of the Study

Our data show a difference in FM among the age groups which peak between 40 and 49 years along with a reduction of FFM around 5^th^ and 6^th^ decade of age. These changes may contribute to the development of sarcopenia, obesity, and osteoporosis at different ages. These conditions share common pathophysiological mechanisms such as insulin resistance, increased levels of proinflammatory cytokines and inflammation, and oxidative stress as well as specific hormonal changes.

This study involved a relatively larger sample size selected randomly from the community. This enhanced the strength of the findings as it reduces the selection bias. The selection criteria used in this study permitted excluding those with abnormal body composition due to prevalent diseases and medication use. All subjects were long-term residents of Galle district and the study area has socioeconomic indices such as poverty, mortality, literacy, life expectancy at birth, and ethnic composition comparable to the entire country according to the data from the Department of Census and Statistics, Sri Lanka (http://www.statistics.gov.lk/). Hence, findings of this study can be generalized to the entire women population in the country and perhaps used as normative reference data of body composition among women aged 20 years and above in Sri Lanka.

Our study, however, has a few limitations such as cross-sectional design and a limited number of women aged 70 years or more. Further, we may have missed some diseases which require detailed laboratory analyses for confirmation. Cohort study design is the more appropriate method to assess age-related changes in body composition as the cross-sectional design tends to overestimate values; therefore, future studies in longitudinal cohort designs are recommended.

## 5. Conclusions

In this study sample, the peak bone and muscle masses are achieved between 40 and 49 years of age. After 50 years, an exponential loss of both bone and muscle masses occurs. FM also peaks between 40 and 49 years of age with a nonsignificant difference over the years. These changes may explain fracture risk, sarcopenia, obesity, and certain diseases seen at different ages.

## Figures and Tables

**Table 1:** Basic characteristics of the participants.

Characteristics	Age category (years)				
	Mean (SD) or frequency (%)				
	20–29 (*n* = 79)	30–39 (*n* = 144)	40–49 (*n* = 185)	50–59 (*n* = 281)	≥60 (*n* = 95)
Age (years)	24 (3)	36 (3)	46 (3)	56 (3)	66 (4)
Age at menopause (years)	—	—	45.5 (2.3) (*n* = 19)	48.7 (3.3) (*n* = 173)	49.6 (3.9) (*n* = 95)
Education level					
** **<Primary education	1 (0.1%)	0 (0.0%)	4 (0.5%)	56 (7.1%)	42 (5.3%)
** **Primary education	7 (0.9%)	65 (8.2%)	26 (3.3%)	113 (14.4%)	21 (2.7%)
** **Secondary education	52 (6.6%)	59 (7.8%)	77 (9.8%)	101 (12.8%)	20 (2.5%)
** **Tertiary education	19 (2.4%)	20 (2.5%)	78 (9.9%)	11 (1.4%)	12 (1.5%)
Income level					
** **<20000LKR	28 (3.6%)	82 (10.4%)	98 (12.5%)	170 (21.6%)	42 (5.3%)
** **20000–50000LKR	23 (2.9%)	55 (70.0%)	81 (10.3%)	92 (11.7%)	23 (2.9%)
** **50000–100000LKR	15 (1.9%)	4 (0.8%)	5 (0.6%)	11 (1.4%)	28 (3.6%)
** **>100000LKR	13 (1.7%)	3 (0.4%)	1 (0.1%)	8 (1.0%)	2 (0.3%)
Total physical activity score (MET/min/week)	4217.52 (1956.02)	5430.59 (2598.09)	6342.43 (3059.11)	6141.44 (2997.91)	4432.67 (2819.73)
Total daily calory consumption (kcal)	1919.45 (612.06)	1710.62 (610.68)	1593.65 (536.56)	1452.33 (814.75)	1862.77 (998.96)
Height (m)	1.54 (0.05)	1.53 (0.05)	1.51 (0.05)	0.49 (0.05)	0.47 (0.05)^*∗*^
Weight (kg)	50.41 (10.90)	57.19 (10.39)^*∗*^	58.30 (10.22)	57.30 (10.68)	53.52 (9.15)^*∗*^
BMI (kg/m^2^)	21.18 (4.25)	24.30 (4.14)^*∗*^	25.25 (4.24)	25.49 (4.21)	24.52 (3.84)
WC (cm)	71.42 (10.04)	80.21 (9.75)^*∗*^	83.62 (10.12)	84.71 (9.70)	93.28 (10.46)^*∗*^
HC (cm)	92.08 (8.89)	96.05 (8.33)^*∗*^	97.63 (8.75)	97.83 (11.29)	95.57 (8.79)
WHR	0.55 (0.15)	0.82 (0.08)^*∗*^	0.84 (0.08)	0.84 (0.11)	0.84 (0.16)

BMI = body mass index, WC = waist circumference, HC = hip circumference, WHR = waist to hip ratio, and LKR = Sri Lankan rupees (1 USD = 200 LKR); ^*∗*^*p* < 0.05 by ANOVA.

**Table 2:** Body composition measurements in different age group.

Measure	Age category (years)				
	Mean (SD)				
	20–29 (*n* = 79)	30–39 (*n* = 144)	40–49 (*n* = 185)	50–59 (*n* = 281)	≥60 (*n* = 95)
TBBMD (g/cm^2^)	1.010 (0.079)	1.033 (0.074)	1.047 (0.084)^*∗*^	0.958 (0.110)^*∗*^	0.882 (0.081)^*∗*^
TSBMD (g/cm^2^)	0.909 (0.114)	0.898 (0.120)	0.919 (0.129)^*∗*^	0.807 (0.143)^*∗*^	0.696 (0.130)^*∗*^
THBMD (g/cm^2^)	0.887 (0.111)	0.899 (0.108)	0.917 (0.124)^*∗*^	0.872 (0.124)^*∗*^	0.796 (0.118)^*∗*^
TBBMC (g)	1657.78 (231.46)	1720.47 (221.56)	1725.42 (225.95)	1507.62 (280.92)^*∗*^	1322.42 (206.09)^*∗*^
TBFM (kg)	18.32 (6.34)	19.78 (5.52)	20.90 (5.74)^*∗*^	21.22 (5.95)	21.25 (5.91)
TBFP (%)	35.86 (6.60)	35.10 (5.13)	35.69 (5.60)	37.02 (94)^*∗*^	38.02 (5.72)
TBLM (kg)	29.72 (4.53)^*∗*^	34.22 (4.91)	34.34^*∗*^ (4.57)	32.99 (4.84)	29.85(3.96)^*∗*^

TBBMD = total body bone mineral density, TSBMD = total spine bone mineral density, THBMD = total hip bone mineral density, TBFP = total body fat percentage, TBBMC = total body bone mineral content, TBFM = total body fat mass, and TBLM = total body lean mass; ^*∗*^*p* < 0.05 by ANOVA.

## Data Availability

The data used to support the findings of this study are available from the corresponding author upon request.
